# Highly flexible and stable resistive switching devices based on WS_2_ nanosheets:poly(methylmethacrylate) nanocomposites

**DOI:** 10.1038/s41598-019-55637-2

**Published:** 2019-12-17

**Authors:** Jeong Heon Lee, Chaoxing Wu, Sihyun Sung, Haoqun An, Tae Whan Kim

**Affiliations:** 1 Department of Electronics and Computer Engineering, Hanyang University, Seoul, 04763 Republic of Korea; 2 College of Physics and Information Engineering, Fuzhou University, Fuzhou, 350108 China

**Keywords:** Engineering, Materials science

## Abstract

This paper reports data for the electrical characteristics and the operating mechanisms of flexible resistive switching devices based on WS_2_ nanosheets (NSs) dispersed in a poly(methyl methacrylate) (PMMA) layer. The ON/OFF ratio of the memristive device based on an Al/WS_2_ NSs:PMMA/indium tin oxides (ITO) structure was approximately 5.9 × 10^4^. The memristive device based on the WS_2_ NSs also exhibited the bipolar switching characteristics with low power consumption and great performance in the bent state with radii of the curvatures of 20 and 10 mm. Especially, the results obtained after bending the device were similar to those observed before bending. The device showed nearly the same ON/OFF ratio for a retention time of 1 × 10^4^ sec, and the number of endurance cycles was greater than 1 × 10^2^. The set voltage and the reset voltage probability distributions for the setting and the resetting processes indicated bipolar switching characteristics. The operating and the carrier transport mechanisms of the Al/WS_2_ NSs:PMMA/ITO device could be explained based on the current-voltage results with the aid of an energy band diagram.

## Introduction

The innovation of information technology has encouraged extensive research into the development of memristive device technologies. The memristive device includes all two-terminal non-volatile memory devices based on resistance switching. From past to now, various types of devices have been fabricated to achieve excellent resistance switching behavior by using various materials such as metal oxide, polymer, and two-dimensional material (2D material) as the active layer^1–3^. Among many alternative memristive devices, resistive switching random access memory (RRAM) devices have received considerable attention due to their relatively simple structure, high integration, low power consumption, and excellent compatibility with complementary metal–oxide–semiconductor (CMOS) technology^4,5^. A typical structure of resistive switching device consists of a composite organic molecule: metal/semiconductor nanoparticle layer sandwiched between two metal electrodes. The device region is defined by the overlap between the upper and the lower electrodes. Therefore, a very high memory density can be achieved by using crossbar arrays^6^. The material used in experiments consists of small inorganic molecules, which have low molecular weights and can be accumulated under high vacuum without thermal decomposition during thermal evaporation^7,8^. However, the attainment of these advantages in practical devices depends significantly on a strong understanding of the resistive switching mechanism, particularly on the atomic level^9–11^. Even though several hybrid nanocomposites have been used to enhance the electrical characteristics of RRAM devices, those materials are still subject of debate because of their uncertainty in different material systems^12^. Thus, the development on stability of RRAM based on reliable materials is critical for the continued optimization and design of this important class of flexible devices^13^.

One of the candidate materials for future RRAMs is tungsten disulfide (WS_2_), which has been intensively studied because of its potential advantages, such as its layer structure, simple composition, ease of fabrication, and high compatibility with CMOS technology^14–16^. In particular, the WS_2_ material has a layer structure that can be easily exfoliated to nanosheets (NSs) by using chemical methods^17^. In the bulk form of WS_2_, the crystal momentums of the minimal-energy state in conduction band and the maximal-energy in the valance band are different (indirect gap). However, these crystal momentums become same (direct gap) when the thickness of WS_2_ is reduced as less than few layers. The bandgap of bulk WS_2_ is 1.3 eV and increases with decreasing number of stacks and reaches a value of 2.05 eV for single layer WS_2_^13,18,19^. This means we can improve the performance of our WS_2_ NSs-based devices by adjusting the bandgap according to the number of layers.

Here, we report flexible memristive devices utilizing WS_2_ NSs:PMMA nanocomposite. The PMMA is used as an insulating dielectric material to transport WS_2_ NSs, taking advantage of its relatively large bandgap compared with embedded WS_2_ NSs. In particular, it improves the memory characteristics by controlling the concentration of WS_2_ NSs, which change on the trap sites in the WS2 NSs:PMMA nanocomposite. The memristive devices utilizing WS_2_ NSs:PMMA nanocomposite perform lower set/reset voltages, larger ON/OFF ratio, longer hold time, and better endurance. Furthermore, the flexible memristive devices utilizing WS_2_ NSs:PMMA nanocomposite also have very high reliability with bent environment.

## Experimental Section

### Exfoliation process for WS_2_ NSs

**Figure 1:**
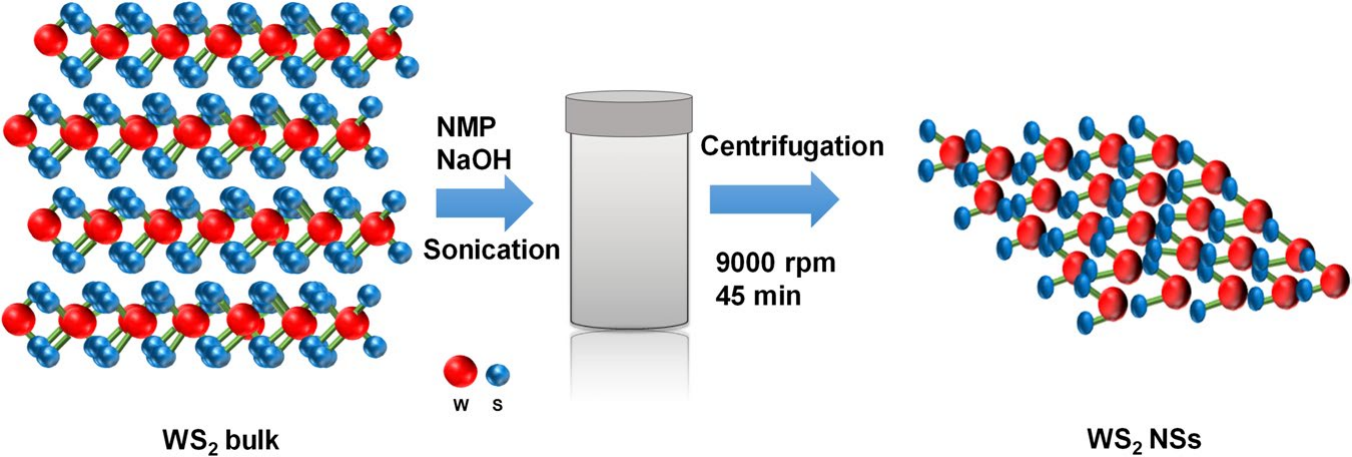
Schematic flow chart of the exfoliation process for the WS_2_ nanosheets.

Figure 1 illustrates the exfoliation process for the WS_2_ NSs. 20 mL of N-methyl-2-pyrrolidone (NMP) was mixed with 0.25 mg/mL of NaOH while varying the WS_2_ concentration from 10 to 50 mg/mL^20^. The mixture was sonicated for 2 h in a sonicator. Cooling during sonication prevented overheating. The mixed solution was put into a high-speed centrifuge for 30 min at 2000 rpm and rotated. The sediment was discarded, and the remaining materials were filtered using a hydrophilic filter. The filtered solution was placed in a centrifuge for 45 min at 9000 rpm. The precipitate was discarded and reintroduced into the sonicator. After sonication for 3 min, centrifugation was performed again at 9000 rpm for 45 min.

### Fabrication of a flexible device based on the WS_2_ NSs:PMMA nanocomposite

For the preparation of the devices, polymethylmethacrylate (PMMA) (average Mw ~ 996000) purchased from Sigma-Aldrich Co. was dissolved in 10 mL of toluene. The stripped WS_2_ was added and stirred at 350 rpm for 9 h by using a magnetic bar so that the solution was sufficiently mixed. A flexible device based on the WS_2_ NSs:PMMA nanocomposite was fabricated with a polyethylene glycol naphthalate (PEN) substrate coated with an ITO electrode. The ITO-coated PEN substrates were sonicated for 20 min using methanol and deionized water, respectively. Thereafter, the cleaned PEN substrates were dried with N_2_ gas, and then subjected to optical treatment with an ultraviolet ozone cleaner for 20 min^2^. A mixed solution of WS_2_ NSs:PMMA was spin-coated onto the ITO-coated PEN substrate for 5 s at 1000 rpm, 10 s at 3000 rpm, 30 s at 5000 rpm, 10 s at 3000 rpm and 5 s at 1000 rpm. The WS_2_ NSs:PMMA nanocomposite layer was heated on a hot plate at 130 °C for 30 min to remove any residual solvent. Thermal evaporation at a chamber pressure of 1 × 10^−6^ Torr was used to deposit a top aluminum electrode with a diameter of 1 mm and a thickness of 200 nm on WS_2_ NSs:PMMA. The blended ratios of WS_2_ NSs in PMMA matrix were varied with 4, 9, 13, and 16 wt% and the memristive devices depending on their WS_2_ NSs blended ratios of 4, 9, 13, and 16 wt% in PMMA matrix were denoted by device I, II, III, and IV, respectively.

### Electrical and physical measurements

As the lower ITO electrode is grounded for each device, a bias voltage applied from the outside is continuously applied to the upper Al electrode. The electrical performance was accurately measured with a Keithley 2400 Digital Source Meter. Scanning electron microscope (SEM) images were obtained using the NOVA NanoSEM 450 system operating at 5 kV. The transmission electron microscopy (TEM) images were obtained by using a CM30 transmission electron microscope at a driving voltage of 300 kV.

## Results and Discussion

**Figure 2:**
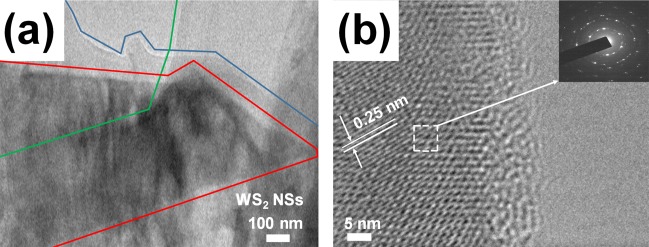
(**a**) Transmission electron microscopy (TEM) image showing a few layers of WS_2_ nanosheets on a carbon TEM grid. (**b**) High resolution-TEM image and an electron diffraction pattern are shown as an inset in the upper right corner.

Figure 2 shows a TEM image of (a) several layered (red line), partially unfolded (green line), and wrinkled sheets (blue line). The WS_2_ are exfoliated by the chemical method described above. Figure 2b is a TEM image of a high-resolution image of the WS_2_ NSs, showing the well-ordered WS_2_, including the insertion of an electron diffraction pattern of WS_2_ NS. This figure shows the border of the end of the sheet and a distinct boundary. A lattice distance measured from the TEM image of the WS_2_ NSs is 0.25 nm, and the inner portion of the WS_2_ NSs have a defect-free crystallographic structure. The electron diffraction pattern shows a six-fold symmetrical structural characteristic of the 2D material and indicates that the crystallized structure of the WS_2_ NSs obtained by the above-described exfoliation method is not destroyed. These results show that the WS_2_ NSs obtained from the above experiment have a good quality^20^.

**Figure 3:**
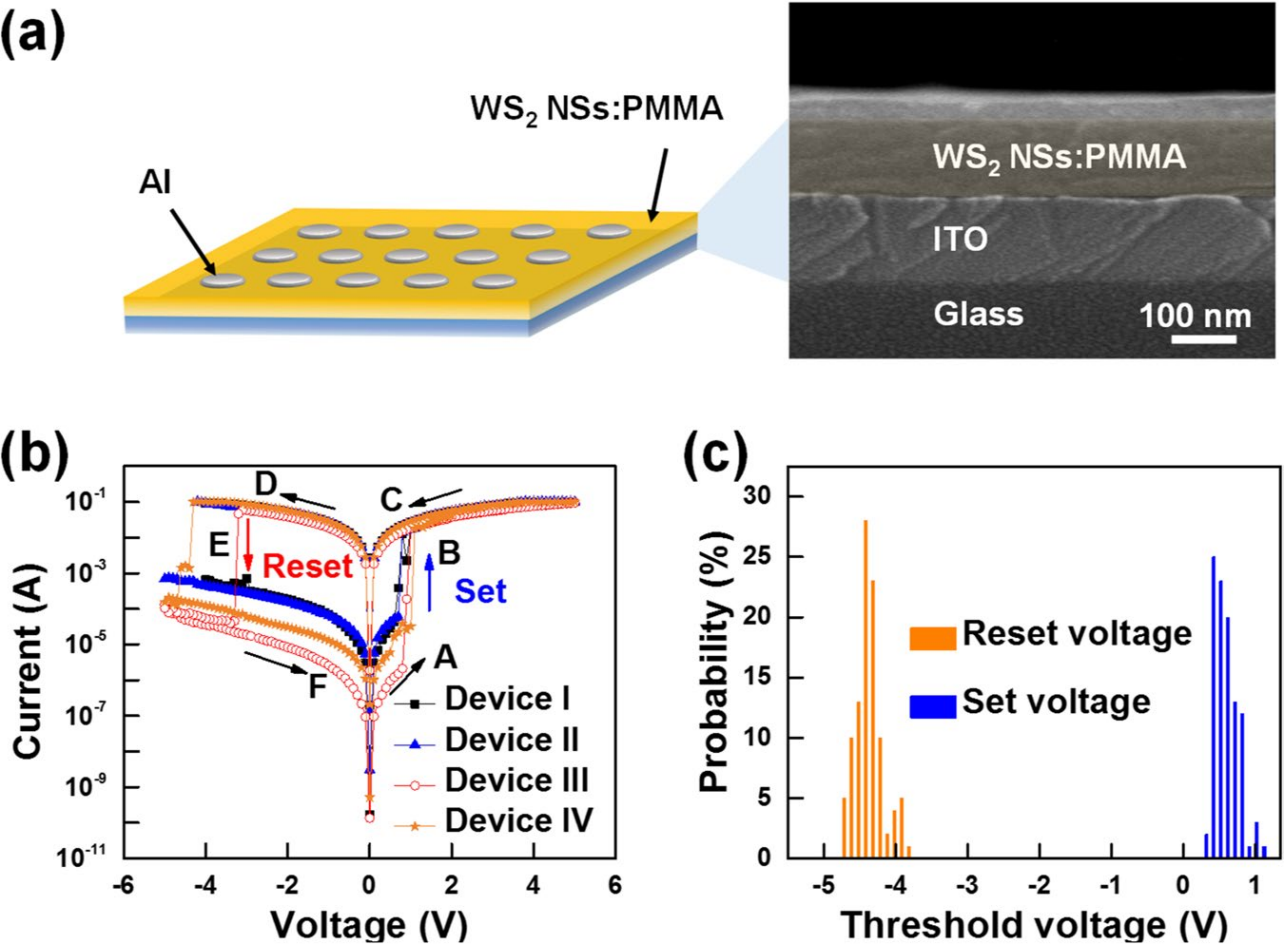
(**a**) Schematic diagram of the fabricated devices with a structure of Al/WS_2_ NSs:PMMA/ITO/Glass and cross-sectional SEM image of the WS_2_ NSs:PMMA layer (highlighted) on an ITO-coated glass substrate. (**b**) I-V curves for the devices I, II, III, and IV with WS_2_ NSs:PMMA nanocomposites. High-resistance state (HRS) and an ‘OFF’ state (Region A, 0 V ~ set), the current suddenly increased with switching to low-resistance state (LRS) and remained there (Region B, set ~ 5 V), LRS and maintains the ‘ON’ state (Region C and D, 5 V ~ 0 V and 0 V ~ reset), the current suddenly decreased with switching to the HRS (Region E, 0 V ~ reset), and HRS and maintains the ‘OFF’ state (Region F, reset ~ 0 V). This result shows that the device has bipolar resistive switching characteristics. (**c**) The probability distributions of the set voltage (V_s_) and the reset voltage (V_r_) from the Device I to Device IV.

Figure 3a shows the schematic diagram for the structure of the memristive devices utilizing WS_2_ NSs:PMMA nanocomposite and a cross-sectional SEM image of WS_2_ NSs:PMMA/ITO/glass. The thickness of the WS_2_ NSs:PMMA active layer is about 100 nm, which is uniformly deposited on the ITO/glass. The PMMA is used as an insulating dielectric material to transport WS_2_ NSs as described above. The cross-sectional SEM image confirms that WS_2_ NSs: PMMA is uniformly deposited in the active layer, so that more reliable results can be obtained when measuring all top electrodes of the device.

Figure 3b shows the current-voltage (I-V) curves of the devices I, II, III, and IV. A bias voltage was applied, with the Al layer serving as the upper electrode and the ITO layer serving as the lower electrode, which was grounded. Clearly, the device III showed the largest memory margin as a switching device because the distinction between the set and the reset region becomes clearer compared that with other devices. Maximum current ratio between ON and OFF states for device III at 0.8 V are equivalent to approximately 5.9 × 10^4^. The ON/OFF current ratio of Device III is 2.45 × 10^2^ times higher than that of Device I. A trap concentration, which is dramatically changed as the concentration of the WS_2_, affects the change in current depending on the voltage applied by the child’s law^21^. However, the ON/OFF current ratio for the device IV is smaller than that for the device III. This is because large agglomerates of WS_2_ NSs are formed between the PMMA molecules^22^. Figure 3c shows the probability distributions of the set voltage (V_s_) and the reset voltage (V_r_) of Devices I, II, III, and IV. The V_r_ values for the devices were dominantly distributed between −4.3 and −4.5 V while the V_s_ values were mainly dispersed between 0.4 and 0.6 V. On the basis of these results, the set and the reset voltages of Devices I, II, III, and IV are 0.5 and −4.4 V, respectively.

**Figure 4:**
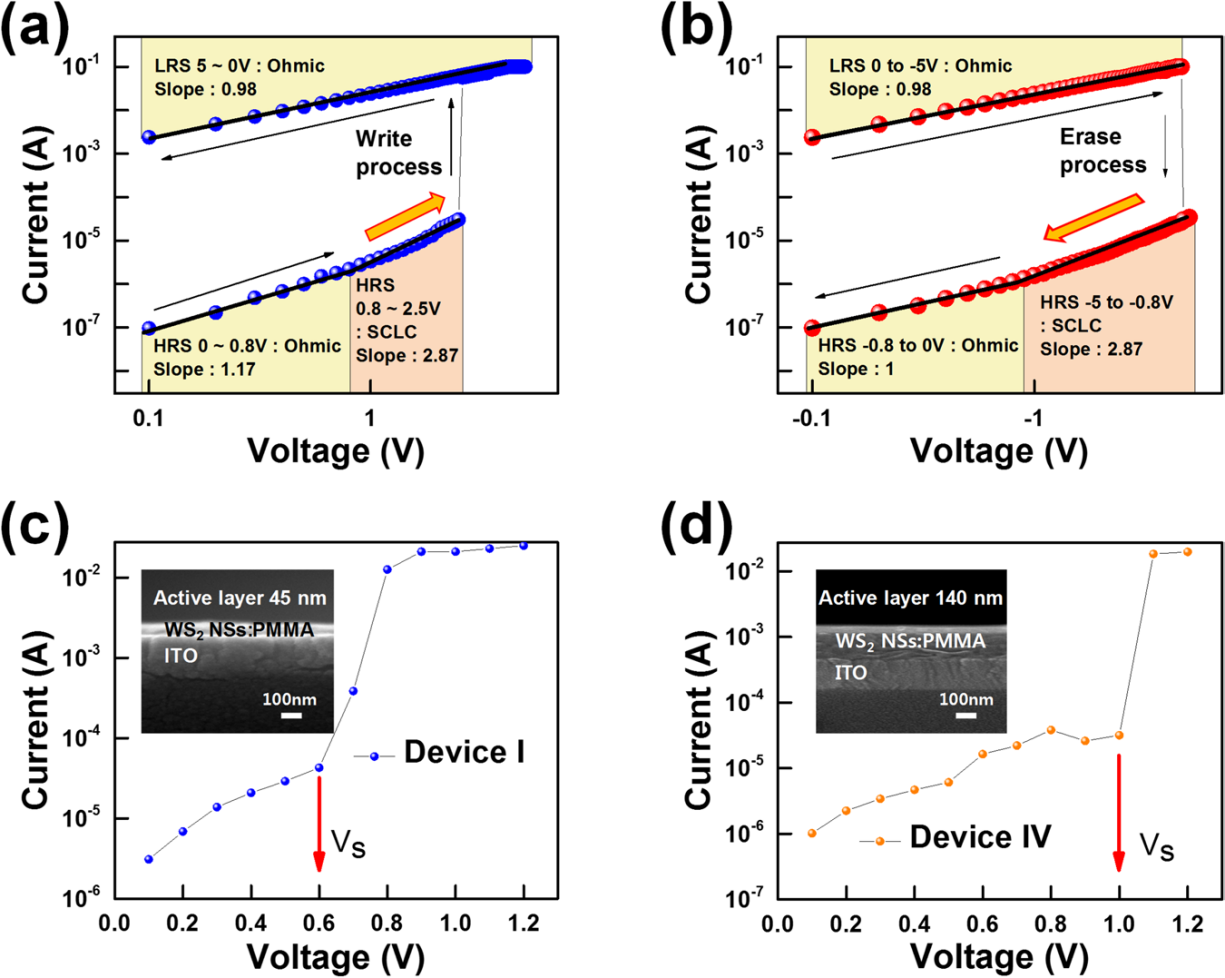
Current-voltage (I-V) fitting curves on a log-log scale to illustrate the carrier transport mechanisms for (**a**) Write process and (**b**) Erase process (Ohmic: J ∝ V SCLC: I ∝ V^2^). (**c**,**d**) The change of set voltage according to the thickness of the device (Device I is 45 nm and Device IV is 140 nm).

The I-V curves in log scale are shown in Fig. 4a,b to address the carrier transport mechanisms at play in the WS_2_ NSs:PMMA-based memristive devices. Figure 4 is obtained by resizing the logarithmic scale of an I-V graph using the Origin Pro 2016 program (Academy version). Because the slope of the fitted I-V curve for the device in the HRS is 1.17, as shown in Region A of Fig. 3, Ohmic conduction behavior is dominant in the HRS. Because the change in the current is approximately proportional to the applied voltage, a good conductive filament probably exists due to electron trapping in the HRS^23^. Then the all traps in active layer is filled with injected electrons in WS_2_ NSs:PMMA became completely occupied by electrons due to space-charge-limited conduction (SCLC) which the current is proportional to the square of the voltage^24^. The slope therefore represents 2.87 in HRS from the I-V curves. As the electric field of the LRS appeared at 2.5 V, which corresponds to “Write process” and it has a slope of 0.98 and maintains the LRS from 5 V to 0 V^23^.

Although negative bias is applied, in Fig. 4b, the slope of the fitted I-V curves is dominant on Ohmic conduction behavior in LRS from 0 to −5 V as shown by the slopes of 0.98. When the electrons in the trap are released, LRS changed to HRS at −5 V as reset voltage, which corresponds to “Erase process”^25^. Since there are still electrons in the trap, it occurs SCLC from −5 V to −0.8 V in HRS as shown by the slopes of 2.87. When the electrons sufficiently escape from the trap, it changes to Ohmic conduction behavior from −0.8 V to 0 V as shown by the slopes of 1. Hence, the carrier transport mechanisms at play in the memristive device based on the WS_2_ NSs:PMMA active layer could be described based on the following models. Figure 4c,d show the set voltages for Devices (c) I and (d) IV with active layer thicknesses of 45 and 140 nm, respectively. The set voltage can be seen to increase with increasing thickness of the active layer. This behavior can be explained by an increase in the number of trap sites with increasing thickness due to more electrons being trapped as a result of the increased set voltage.

**Figure 5:**
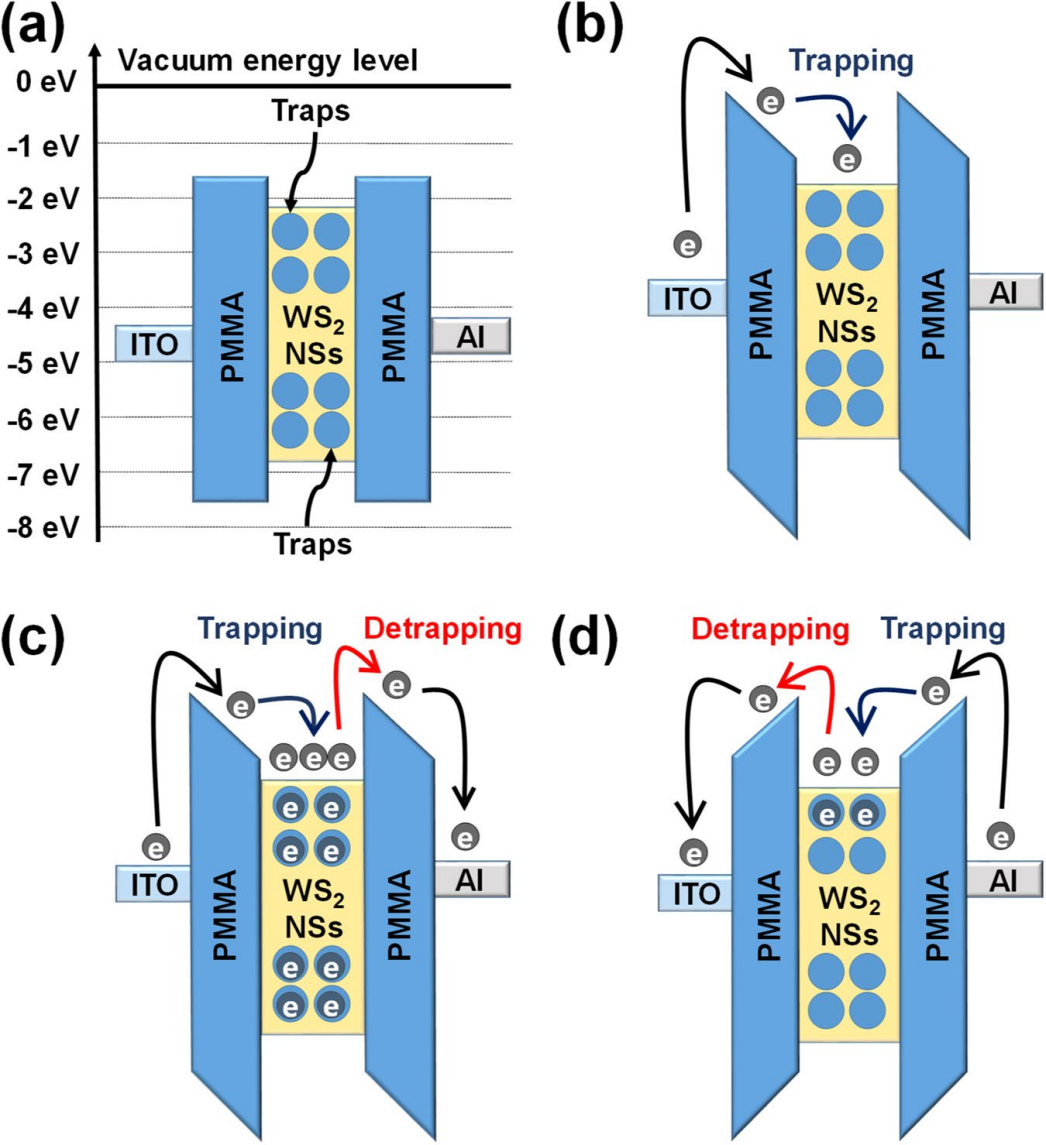
(**a**) Schematic diagram of the energy-level bands for the WS_2_ NSs:PMMA/ITO/PEN memristive device without applied bias. Schematic diagrams of the carrier transport mechanisms in the LRS writing processes when (**b**) a low bias voltage (0–0.8 V) and (**c**) a high bias voltage (0.8–2.5 V) are applied. (**d**) Schematic diagram of the carrier transport mechanisms in the HRS erasing process. The work functions of the ITO film and the Al electrode are −4.8 and −4.3 eV, respectively. The highest occupied molecular orbital level of PMMA is −7.8 eV, and the lowest unoccupied molecular orbital level is −1.8 eV.

The carrier transport behaviors and the mechanisms for the set/reset operations of memristive devices with their energy band diagram are described in Fig. 5a–d^26^. The electron injection efficiency from the ITO electrode is relatively high compared to that from the Al electrode, as shown in Fig. 5b^27,28^. When the device at low bias voltages between 0 and 0.8 V is in the HRS, the current linearly increases with increasing applied voltage. This result indicates that relatively few carriers occupy WS_2_ NS sites, as shown in Fig. 4a. When high bias voltages between 0.8 and 2.5 V are applied, because the electrons existing in the ITO electrode can overcome the energy barrier, they move to the WS_2_ NS sites via the Schottky emission process^29^.

**Figure 6:**
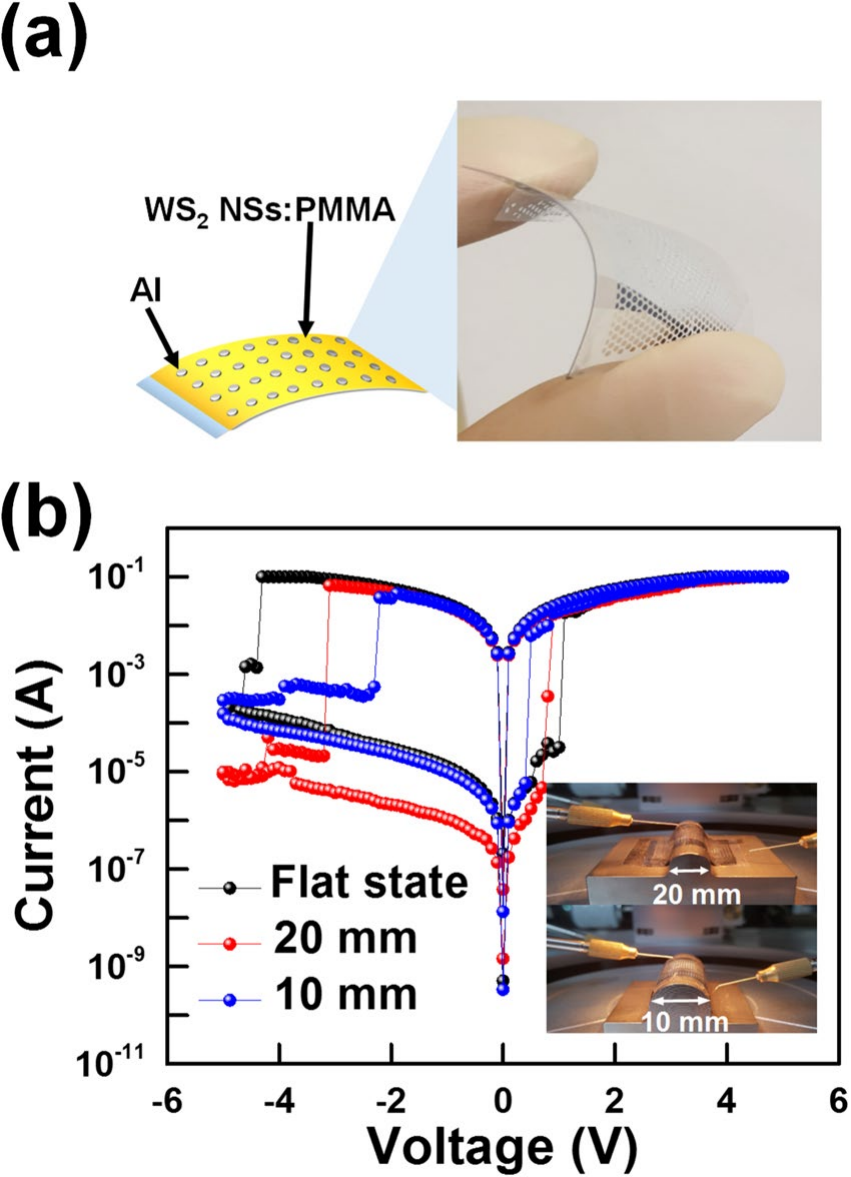
(**a**) Schematic diagram and photograph of the devices with a structure of Al/WS_2_ NSs:PMMA/ITO/PEN. (**b**) ON-state currents of the device IV in the flat and in the bent states with radii of curvature of 20 and 10 mm and a photographs of the devices are shown as an inset in the bent state with radii of curvature of 20 and 10 mm.

The electrons injected from the ITO electrode are transferred to the WS_2_ NS sites along the direction of the electric field generated by the applied voltage. In Fig. 5c, The WS_2_ NSs are seen to act as trapping sites due to the conduction-band energy difference between the PMMA layers. Thus, the space charge formation resulting from the electrons trapped in the sites dominates the conduction process^30,31^. When the device changes from the HRS to the LRS, the current in the device remains constant until a negative voltage is applied to the device, which is indicative of a nonvolatile memory behavior. When low bias voltages between 0 and the reset voltage are applied, even though the electrons begin to escape from the traps, the device remains in the LRS. When a high negative bias is applied to the device, Poole-Frenkel (P-F) emission occurs^32^. When a high negative bias is applied to the upper Al electrode, a detrapping process from the sites occurs, and some electrons are emitted from the potential wells, as shown in Fig. 5d^33^. As a result, the current is significantly reduced, and the device returns to the HRS, which corresponds to an erase process in the memory^23,30^. Figure 6 shows the results of I-V characteristics when the device is bent. Figure 6a shows the structure and photograph of the Al/WS_2_ NSs:PMMA/ITO/PEN device. The PEN substrate was chosen for testing in a flexible environment instead of glass as a bottom substrate. Figure 6b shows the I-V curves of the device IV in the flat state and in the bent state with radii of curvature of 20 and 10 mm^34^. The current of the LRS for the device with a radius of curvature of 10 mm was about 1.4 times smaller than that for the device with a radius of curvature of 20 mm. When the device is bent, the ITO electrode becomes strongly stressed, and as the bending radius of the device decreases, a few cracks start to appear in the ITO^35^. The crack density of the ITO increases with decreasing bending radius, resulting in increased resistance^36^. Therefore, the programming and the erasing voltages decrease with decreasing radius of curvature (bending radius) of the device, and the current varies as shown in Fig. 6b.

However, there is no critical damage and distortion in the operation of the memristive devices in a flexible environment. As the device with a radius of curvature of 10 mm has an on/off ratio up to 10^3^ at 0.5 V, the device composed of WS_2_ NSs:PMMA material has a memory characteristic despite being a device bent.

**Figure 7:**
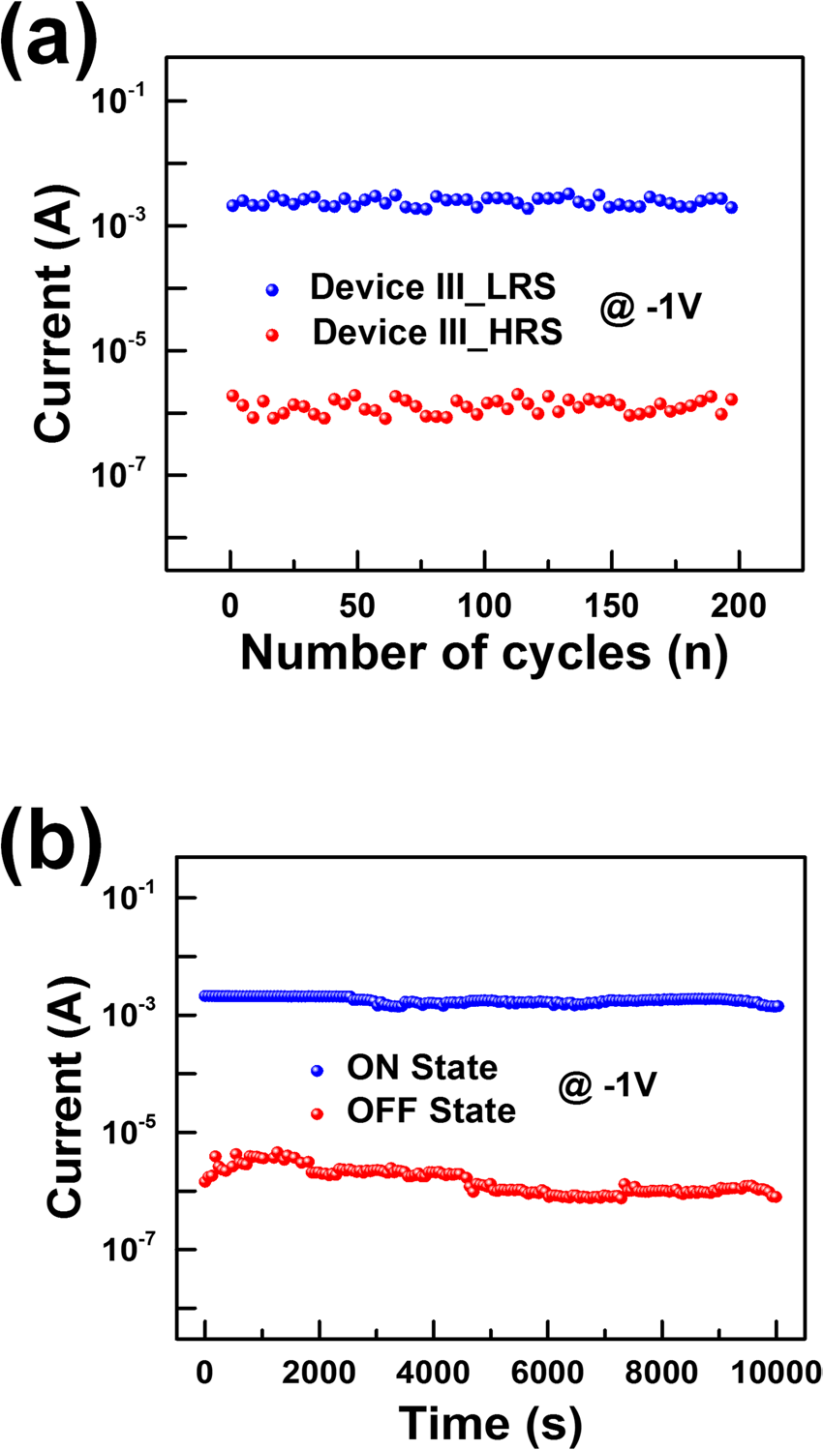
(**a**) Endurance characteristics of the device III after bending with a radius of curvature of 10 mm. (**b**) Retention characteristics of the device III after bending with a radius of curvature of 10 mm.

Figure 7 shows stability and reliability data for our device when operated for a long period of time. Device III was selected to clarify the difference between the HRS and the LRS. Figure 7a shows the endurance capacities of the HRS and the LRS measured after bending with a radius of curvature of 10 mm under a readout voltage of −1 V. No significant changes in the LRS/HRS ratio can be seen during 200 bending cycles. The retention time data in Fig. 7b shows the reliability of our device for lengthy operations after bending with a radius of curvature of 10 mm. The ON and the OFF states of the WS_2_ NSs:PMMA-based device were maintained at 2.6 × 10^−3^ A and at 6.1 × 10^−7^ A, respectively, with no significant change being observed for a maximum of 10^4^ seconds.

## Conclusions

In summary, WS_2_ NSs are promising materials for building high-performance and flexible RRAM devices. I-V curves, I-V fitting curves, and schematic diagrams were used to illustrate the principle of operation of devices with WS_2_ NSs. The devices based on the WS_2_ NSs:PMMA nanocomposite exhibited significant resistance-switching memory performance, including low operating voltages (0.8 V), large resistance ON/OFF ratios (>10^4^) and long retention times (>10^4^). This RRAM device based on WS_2_ NSs:PMMA nanocomposite also demonstrated excellent flexibility due to their 2D characteristics. In particular, the devices based on the WS_2_ NSs exhibited great performance in the bent state with radii of the curvatures of 20 and 10 mm. These results mean that WS_2_ NSs-based flexible resistive switching devices are suitable for applications in next-generation wearable devices.
